# Simulating the integration and regulation of human Ia and Ib reflexes on a musculoskeletal robot driven by pneumatic artificial muscles

**DOI:** 10.3389/frobt.2026.1741690

**Published:** 2026-02-18

**Authors:** Junqi Wang, Ryu Takahashi, Yiqi Li, Yelin Jiang, Koh Hosoda

**Affiliations:** 1 Graduate School of Engineering, Kyoto University, Kyoto, Japan; 2 Graduate School of Engineering Science, Osaka University, Osaka, Japan

**Keywords:** bio-inspired robot, double-reflex control system, local reflex control, musculoskeletal robot, pneumatic artificial muscles, robotics

## Abstract

Simulations of human Ia and Ib reflexes on a bio-inspired musculoskeletal robot driven by pneumatic artificial muscles (PAMs) offer a favorable option for counteracting disturbances in complex and dynamic work environments, providing a solution to the significant computational burdens that undermine its potential due to PAMs’ inherent non-linearities. This research focuses on the simultaneous integration of human Ia and Ib reflexes (referred to as double-reflex) as countermeasures against physical disturbance in a musculoskeletal robot system driven by PAMs. The system’s performance was examined, and implementation challenges were identified during experiments. Mechanisms were then applied to ensure the effective functioning of the integrated reflexes. Experimental results substantiated the effectiveness of the double-reflex system, highlighting its functionality within the robotic system. This investigation corroborates the viability of concurrently implementing Ia and Ib reflexes, providing a reference for the design of robotic reflex control systems. The study also offers some references based on the view of signal processing, regarding the possible functions of the human spinal cord that might be necessary to perform proper reflex actions.

## 1 Introduction

The bio-inspired musculoskeletal robot exhibits greater robustness, flexibility, and redundancy compared to traditional motor-driven robotic systems (Wu et al., 2021; Tondu and Lopez, 1997; Daerden and Lefeber, 2002; Tondu et al., 1994). Unlike general motor-driven robots, which are mostly designed for pre-programmed tasks within controlled environments, the musculoskeletal robot demonstrates higher adaptability when executing various tasks within complex and dynamic environments (Lee and Shimoyama, 1999). Such adaptability indicates significant potential for bio-inspired musculoskeletal robots to be utilized in human society, which usually requires them to maneuver through intricate urban landscapes. Several types of musculoskeletal structures have been developed by previous research studies in the field to mimic the human muscle structure (Newman, 1989; Weng and Young, 1996; Bicchi and Tonietti, 2002; Tonietti and Bicchi, 2002; Anh et al., 2018; Hannaford et al., 1995; Wu et al., 1997; Eskiizmirliler et al., 2002; Ouanezar et al., 2011; Ijspeert, 2008).

As one of the most widely-recognized forms of pneumatic artificial muscles (PAMs), the McKibben artificial muscle was used by these designs due to its straightforward design, ease of implementation, and similar behavior to skeletal muscles (Tondu, 2012). However, the high non-linearities of the McKibben structure also lead to poor dynamic accuracy, making precise control very hard to model and realize. Traditional control models intended for McKibben-driven musculoskeletal robots tend to be extremely complex, and therefore, they impose heavy computational pressure on the processing unit (Vanderborght et al., 2006; Chikh et al., 2010; Erin et al., 2016; Ahmad Sharbafi et al., 2017).

In order to alleviate the computational pressure of the pneumatic-driven musculoskeletal robot under numerous interferences, a local reflex control system was proposed as a new control method. Inspired by human spinal reflexes (Kandel et al., 2000), the local reflex control system will react to unexpected interference applied to the robot with pre-programmed feedback actions independently. In the study by Takahashi et al. (2023), a local reflex control system was developed to emulate two primary human type-I peripheral reflexes, namely, the Ia and Ib reflexes. The study confirmed the role of reflexes in enhancing stability under external disturbances.

The prior research did not investigate the system’s behavior when both reflexes were implemented concurrently, nor did it present solutions for managing post-reflex disturbances. The system was designed to stop immediately upon the completion of a single reflex action and was unable to return to or evade its pre-reflex state, depending on the reflex type. The research also indicated that post-reflex disturbances could cause greater positional overshoots than the initial disturbances, posing significant challenges for both the system stability and the coexistence of multiple reflexes in a single trial.

This study examines the simultaneous implementation and performance of both Ia and Ib reflexes within the musculoskeletal robotic system. A modified version of the one-degree-of-freedom musculoskeletal system proposed by previous research was used to test the combined reflex control on specific motor tasks. Subsequently, additional mechanisms were integrated into the control system during experiments to address emerging challenges during reflex integration.

This research yields results that demonstrate the performance of the double-reflex system. After the discovery of several problems when integrating two reflexes in practice, a set of example mechanisms that regulate and smooth double-reflex actions was proposed as a reference for future reflex-system designs.

## 2 Double-reflex system

This research inherits the basic setup of the one-degree-of-freedom (DOF) musculoskeletal system proposed by Takahashi et al. (2023). Since the original design was built for testing Ia and Ib reflexes separately, the system lacks any mechanism to ensure that Ia and Ib reflexes can function effectively with each other, and it stops immediately after a single reflex feedback action is completed. Thus, several modifications were made to ensure that the double-reflex system can function smoothly.

### 2.1 General work flow

The system utilizes the same artificial muscle spindle sensor (AMS) and artificial Golgi tendon organ sensor (AGTO) presented by Takahashi et al. (2023) to trace the length and force load on the muscles.

**FIGURE 1:**
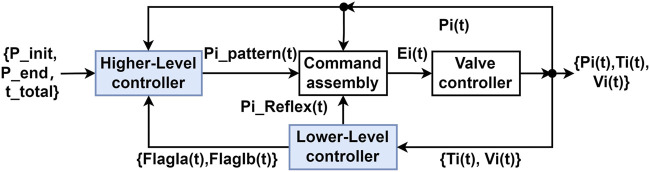
Schematic diagram of the double-reflex system. The blue blocks in the diagram indicate the modules that contain newly implemented mechanisms.

Figure 1 shows the general flow of the signal processed by the system. Similarly to the original design, the higher-level controller generates a set of pattern pressures based on the initial pressure $P_{\mathrm{init}}$ , end pressure $P_{\mathrm{end}}$ , and function time range, which are pre-defined. The pattern pressures are then be passed into the double-reflex system, which can be considered a combination of the lower-level controller and command assembly. Next, the goal pressures are be summed up with triggered reflex/co-contraction feedback generated from the lower-level controller and output an assembled goal pressure command to calculate the error pressure for the valve controller. Then, the PID controller within innervates the proportional values to carry out the pressure adjustment of the PAMs. Notably, the pattern generator in the higher-level controller is not a part of the local-reflex system design and can be replaced by any other pattern generation method. The presented one merely provides the minimum function to enable the system to conduct meaningful tasks.

### 2.2 Higher-level controller

**FIGURE 2:**
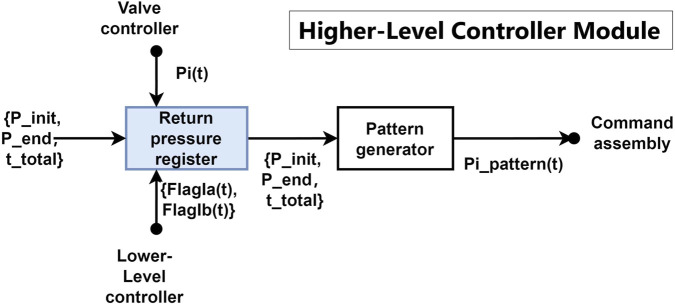
Schematic diagram of the higher-level controller. The blue blocks indicate the newly implemented mechanisms to achieve the double-reflex system.

Although the higher-level controller is not part of the system on which we mainly focused, a new function capable of tracking the pressure state of the PAMs is necessary to ensure that the system returns to its motor task after reflex feedback. The detailed schematic of the modified higher-level controller is shown in Figure 2.

The newly added return pressure register shown in the figure saves the pressure readings from PAMs before or after the reflex feedback, depending on the triggered reflex type. It receives and interprets the status of the reflexes’ feedback via the flag signals FlagIa(t) and FlagIb(t) sent from the lower-level controller. In the case of the Ia reflex (stretching reflex), the goal is to prevent the muscles from stretching too quickly, which is mostly caused by sudden impacts, and the system is expected to return to its original position afterward. Therefore, the pressure reading right before the Ia reflex is recorded in the return pressure register. The Ib reflex, unlike the Ia reflex, aims to prevent the muscles from becoming overloaded. When the Ib reflex is triggered, the system tends to move away from the muscle’s current pressure at the moment of triggering in order to alleviate overload. Thus, the pressure reading immediately after the Ib reflex is recorded in the return pressure register. After each triggered reflex, the recorded pressure is sent to the pattern generator and becomes the new $P_{\mathrm{init}}$ for the remaining trial period. If no reflex is triggered, the pattern generator remains in its original setting, outputting the pattern pressure $P_{pattern\left(t\right)}$ and sending it to the command assembly module.

Any pattern generator with the ability to track the real-time joint position and update the goal pattern command can be used to replace the presented module.

### 2.3 Lower-level controller

As the most important part of the double-reflex system, the lower-level controller is responsible for generating the reflex pressure command based on the sensory inputs from AMS and AGTO within the valve controller. The previous system only begins capturing and recording signal inputs once the threshold is reached. In contrast, the new system is continuously listening and always updates the highest signal inputs, even before the threshold is reached. This leads to unstable feedback action to the exact same outer interference. To resolve this limitation, three new mechanisms were introduced, namely, Max tracker, co-contraction, and mutex lock.

**FIGURE 3:**
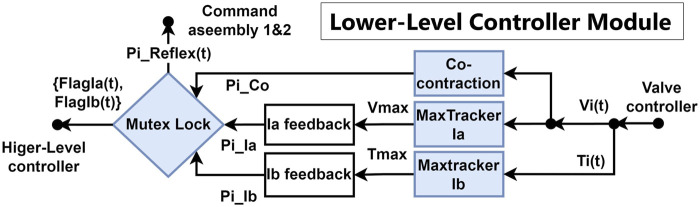
Schematic diagram of the lower-level controller. The blue blocks indicate the newly implemented mechanisms to achieve the double-reflex system.

To counteract the inherent delay and non-linearity of the McKibben muscle, the Max tracker Ia/Ib shown in Figure 3, which denotes the maximum signal spike tracker Ia/Ib, continuously updates the highest signal inputs from AMS and AGTO throughout the motor task. When a reflex action is triggered, the saved signal is used to calculate the feedback pressure, and it is reset to zero upon completion of the reflex action. The maximum inputs are then modulated with the empirical factors $K_{Ia}$ and $K_{Ib}$ and become the reflex pressure commands:

$$P_{i\text{\_}Ia}=K_{Ia}\times V_{\max},$$ (1)

$$P_{i\text{\_}Ib}=K_{Ib}\times T_{\max}.$$ (2)

The calculations were done with Equations 1, 2, $K_{Ia}$ and $K_{Ib}$ are the empirical factors that are determined by the system’s performance in the preliminary experiments.

By mimicking the way humans increase limb stiffness to achieve precise control (Lewis et al., 2010; Osu et al., 2002; Yeadon et al., 2010; Milner and Cloutier, 1998; Büchler et al., 2023), co-contraction was integrated into the lower-level controller as a damper to counter post-reflex interference. It functions as a special sub-reflex that also uses stretching speed as its input reference to ensure a fast response. Co-contraction is designed to occur before and after reflex feedback to suppress long-term oscillations of the system. The magnitude of the co-contraction feedback is set as a constant 10% of the maximum pressure output.

The pressure signals of the Ia reflex, Ib reflex, and co-contraction are then processed by the mutex lock module, which is inspired by the similar function of the human spinal cord (Lundberg, 1979). The mutex lock module ensures that the system can perform a complete reflex feedback action. It blocks the system from receiving certain sensory inputs during the reflex feedback period, preventing rapid and strong reflex and post-reflex behaviors from driving the system into an endless mis-triggering loop. The mutex lock module can also be customized to prioritize the Ia and Ib reflexes and co-contraction. In this research, the Ia reflex cannot be triggered during Ib reflex feedback since it poses a risk of overloading the system again; however, it can be triggered during co-contraction feedback once the set threshold is reached. The priority of the feedback can be considered to be Ib > Ia > co-contraction with this setting. After passing through the mutex lock, the pressure commands are sent to the command assembly module of both PAMs as $P_{1\text{\_}Reflex}\left(t\right)$ and $P_{2\text{\_}Reflex}\left(t\right)$ . A pair of Boolean flag signals FlagIa(t) and FlagIb(t) will also be sent to the higher-level controller to indicate the current status of the reflex feedback.

The lower-level controller module can be deployed independently on any joint driven by an antagonistic PAM muscle group as a local reflex controller. Notably, while the Max tracker mechanism can be applied directly, the co-contraction and mutex lock mechanisms will require re-tuning of their parameters to fit the PAM characteristics and the requirements of the specific motor task.

### 2.4 Command assembly

**FIGURE 4:**
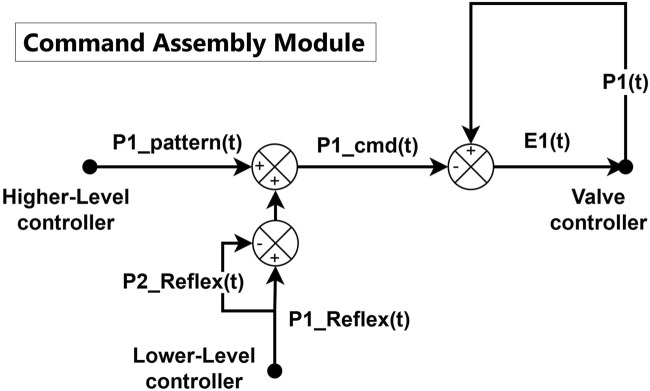
Schematic diagram of the command assembly for PAM1. The PAM2’s command assembly module is symmetric to the shown diagram.

Figure 4 shows the schematic diagram of the command assembly module of PAM1, the agonist PAM of the system, which is symmetric to the command assembly module of PAM2, the antagonist PAM. The command assembly module is responsible for summing up all pressure commands from the higher-level and lower-level controllers. Additionally, the reflex pressure command from the opposite side ( $P_{2\text{\_}\text{Reflex}\left(t\right)}$ in this case) is also considered in the assembly, mimicking human reciprocal excitation and inhibition. The final command $P1_{\mathrm{cmd}}$ is then calculated and compared with the current pressure $P1_{t-1}$ (before reflex feedback) to determine the error pressure that is then passed into the valve controller module. The calculation can be summarized as Equation 3:

$$E_{1}\left(t\right)=P_{1\text{\_}Pattern}\left(t\right)-P_{2\text{\_}Reflex}\left(t\right)+P_{1\text{\_}Reflex}\left(t\right)-P_{1}\left(t-1\right).$$ (3)

The command assembly module is a purely software-based module that combines the pattern pressure and the reflex/co-contraction feedback pressure based on the needs of the motor task and sends the result to the valve controller for PID control. In this study, we present a simple summation method for command assembly in a 1-DOF system, but more complex behavior can be achieved by adding additional contribution factors, such as gravity compensation and coordinated pressure commands from other connected joints.

### 2.5 Valve controller

The valve controller is the straight-forward action part of the system. It carries out the valve actions based on the error pressure command from the command assembly module and returns the pressure, tension, and rate of length change from the pressure sensor, AGTO, and AMS, respectively, to the modules that require them.

### 2.6 Specific motor task

**FIGURE 5:**
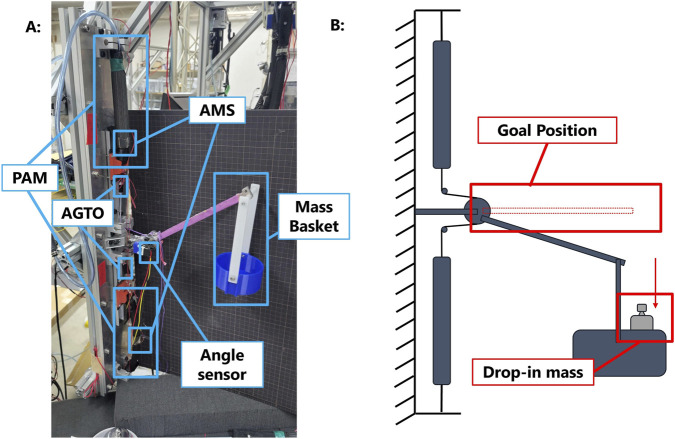
Overview of the double-reflex system. **(A)** Hardware setup of the double-reflex system and **(B)** design of the specific motor task for the double-reflex system.

This study aims to evaluate the performance of the reflex system in realistic scenarios, such as when an impact object remains in contact with the system and continues to apply gravitational force. Thus, in the specific motor task design, the system was tasked with maintaining the lever in the middle position, where both muscles operate at 50% of the maximum pressure output (Figure 5B). The deviation of the lever’s angle caused by the system’s own weight was auto-remapped to zero at the beginning of every trial. The total mass is dropped from the connection between the basket and the lever into the basket when the lever is in the horizontal position. A mass of 200 g was dropped into the basket to trigger the Ia reflex. Following this reflex, a 500-g mass was gently added to the basket to trigger the Ib reflex.

### 2.7 Hardware setup

As shown in Figure 5A, the basic part of the system remains the same as in the original design: two McKibben PAMs are fixed at one end to either side of the platform, while their other ends are attached to track sliders to ensure free movement along the track. The AMS are wrapped around the two PAMs to measure changes in diameter, which are proportional to changes in length. The AGTOs are then connected to the sliders with fishing lines to measure the tension force on both sides. The fishing line is tied to the central pulley, where the lever and angle sensor are attached.

To assess the performance of the double-reflex system in a more realistic circumstance, the physical setup was changed in the vertical direction to simulate the working environment of a human upper limb under the effect of gravity. Masses of different weights were used to simulate external impacts. A basket holding the mass was attached to the system’s lever, enabling continuous application of gravitational force even after the initial impact.

## 3 Experiments

### 3.1 Empirical parameters of reflex and co-contraction

Table 1 displays the adjusted parameters and the newly introduced co-contraction parameters used to configure the double-reflex system. Since the system utilizes only analog signals for control, all the parameters in Table 1 were empirically determined through multiple trials.

**TABLE 1:** Empirical parameters of the double-reflex system.

Parameter	Name	Value
$K_{Ia\text{\_}agonist}$	Factor of agonist Ia feedback	1/2000
$K_{Ia\text{\_}antagonist}$	Factor of antagonist Ia feedback	1/200
$K_{Ib}$	Factor of both Ib feedback	1/100
$C_{co-contraction}$	Magnitude of co-contraction feedback	0.05 (MPa)
$V_{ago\text{\_}thres}$	Threshold of agonist stretch speed	260%
$V_{anta\text{\_}thres}$	Threshold of antagonist stretch speed	10%
$C_{ago\text{\_}thres}$	Threshold of agonist co-contraction	130%–260%
$C_{anta\text{\_}thres}$	Threshold of antagonist co-contraction	5%–10%
$T_{\mathrm{thres}}$	Threshold of tension force	4,000
$t_{mutex\text{\_}Ia}$	Mutex lock time period of Ia reflex	0.6 (seconds)
$t_{mutex\text{\_}Ib}$	Mutex lock time period of Ib reflex	0.6 (seconds)
$t_{mutex\text{\_}C}$	Mutex lock time period of co-contraction	0.6 (seconds)

The pressure range for the system’s proportional valves spans from 0 MPa (fully deflated) to 0.5 MPa (fully inflated). The magnitude of the co-contraction feedback is set at a constant $C_{co-contraction}$ of 0.05 MPa, representing 10% of the maximum pressure output. The factors for agonist and antagonist Ia feedback, $K_{Ia\text{\_}agonist}$ and $K_{Ia\text{\_}antagonist}$ , differ due to variations in resistance in handmade AMS sensors. To accommodate these differences and counteract the effect of gravity, the thresholds for agonist and antagonist stretch speed, $V_{ago\text{\_}thres}$ and $V_{anta\text{\_}thres}$ , were adjusted. The co-contraction mechanism also uses the stretching speed of the AMS sensor as thresholds. Additionally, the stretch speed signals and thresholds were converted into percentages of deviation to facilitate smoother analysis. Analog readings from both AMS were scanned before each system start and recorded as baseline values to avoid errors caused by hardware offsets, such as the accumulative shift of the starting position due to elongation of the fishing line under constant tension. For example, the speed threshold of the agonist Ia reflex was calculated via Equation 4:

$$\begin{array}{ll} V_{ago\text{\_}thres} & =\left(V_{ago\text{\_}thres}-V_{base\text{\_}ago\text{\_}speed}\right)/V_{base\text{\_}ago\text{\_}speed}\times 100\%. \end{array}$$ (4)

The thresholds were tuned to ensure that a 200-g mass drop triggers the Ia reflex, while a mass load of 700 g triggers the Ib reflex. Therefore, the impact needed for and generated by the reflexes will not damage the sensors and actuators, and all feedback actions can be triggered separately to facilitate smooth debugging. The mutex lock time periods of Ia and Ib and the co-contraction reflexes were set to 0.6 s.

### 3.2 Performance experiments of the “raw” double-reflex system

The return pressure register and Max tracker were implemented into the original system as a first attempt to address two obstacles identified in previous research: the lack of post-reflex behavior regulation and the unstable reflex feedback magnitude caused by inherent delay. During the experiments, the raw system exhibited unstable and random behaviors. The system would either enter a continuous oscillation until manually stopped or provide only insignificant feedback in response to the initial impact caused by a mass drop.

**FIGURE 6:**
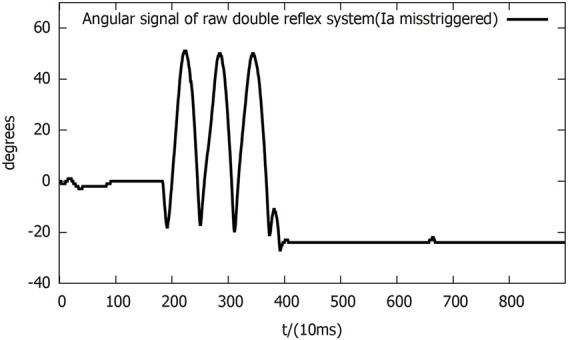
Angular signal of the raw double-reflex system in Ia reflex mis-triggering.

As shown in Figure 6, the initial impact occurred at approximately 2,000 ms, triggering the first Ia reflex, as represented by the first peak in the angular signal plot. The rapid fall of the lever created a second impact on the AMS and triggered the second Ia reflex in the same direction. After one more similar mis-triggering, the threshold of the opposite AMS was breached, and the feedback action was triggered in the opposite direction. Notably, the maximum negative angle is smaller than the maximum positive angle because the height of the mass basket reduced the clearance it can drop.

**FIGURE 7:**
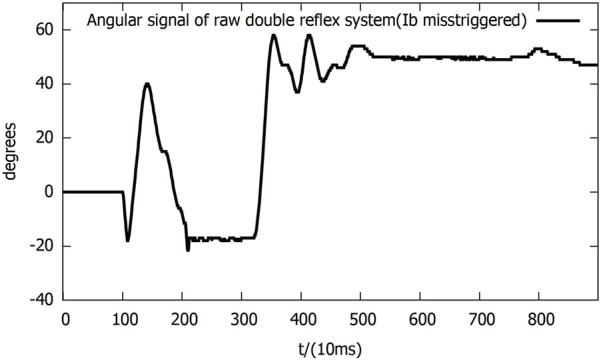
Angular signal of the raw double-reflex system in Ib reflex mis-triggering.

As shown in Figure 7, the initial impact triggered the Ia reflex, with the help of gravity, and the Ib reflex threshold was breached when the lever fell down. The lever was then lowered and locked in its lowest position. After approximately 1,000 ms, the reverse Ib reflex was triggered due to system escalation, returning the lever to its high position and maintaining it there. This occurred because the Ib mis-trigger caused the return mechanism to function incorrectly, as the mechanism was not designed to account for an Ib mis-trigger.

From these experiments, it is clear that the simple threshold system from the original design cannot recognize the impact caused by the reflexes and, therefore, falls into an endless loop of mis-triggering; a new reflex will be triggered even before the last one is finished. This causes the reflex to become an even greater interference with the system, defeating the purpose of the local reflex-control system.

Two conclusions can be derived from this problem: the threshold of the reflex should not be set too close to the maximum capacity of the PAMs since the feedback will have an equal or greater magnitude than the initial impact, and a faster speed of recovery is achieved by greater oscillation in the short term; to make two reflexes work together, we must first “separate” them properly in terms of signal processing. Additionally, although the Max tracker mechanism ensures that the system provides stable reflex feedback, the system will still occasionally generate feedback with extremely small magnitudes. This phenomenon is caused by mutual cancellation, as the feedback signals are triggered simultaneously in opposite directions and cancel each other out in the command assembly module.

### 3.3 Performance experiments of the double-reflex system with mutex locks

**FIGURE 8:**
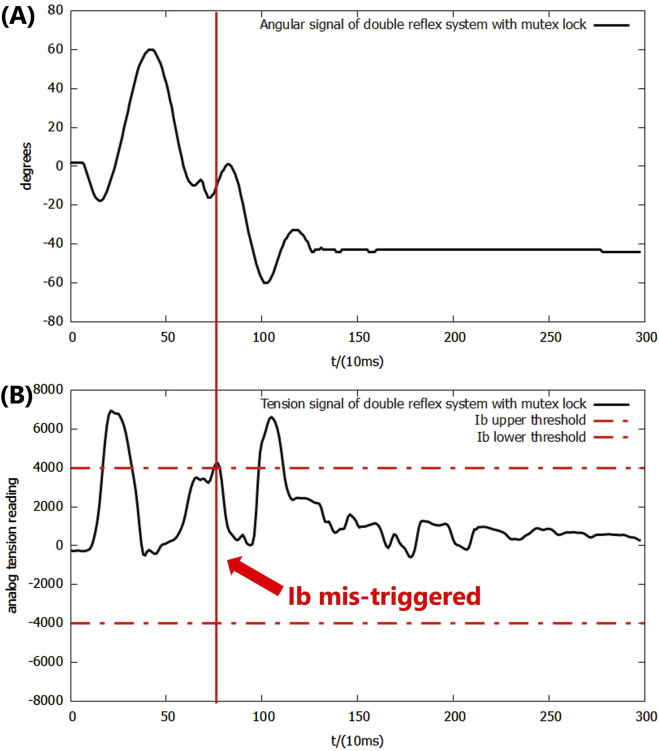
**(A)** Angular signal of the double-reflex system with mutex lock. **(B)** Analog tension signal of the double-reflex system with mutex lock.

In the following experiments, the mutex lock mechanism successfully prevented mis-triggering within the reflex feedback period and the insignificant feedback as it blocked additional reflexes during an ongoing reflex feedback period. However, the mis-triggering between the Ia and Ib reflexes after the feedback period still existed, and all of the mis-triggerings occurred in the same order, where the Ia reflex was always triggered before the Ib reflex (Figure 8A).

By examining the signal inputs of the system (Figure 8), the cause was identified as the unregulated post-reflex behavior: since the Ia reflex against an impact created by a dropped mass always leads to an upward bounce and overshoot, the combined effect of the post-reflex return action and gravitational force generates an equal or greater second impact, which triggers the Ib reflex when the lever drops. Simply elongating the mutex lock time period can cause the system to omit the overloading signal and prevent mis-triggering, but it significantly slows down the overall reflex speed. The post-reflex behavior of the Ib reflex was also unregulated, but its negative effect was avoided by the returning mechanism, which returned the lever to its position after the Ib reflex made the system drop the basket directly to the ground. The identical order of the Ia reflex before the Ib reflex throughout the experiments was very likely caused by the inherent delay of McKibben PAM and differences in how the AMS and AGTO sensors operate. This ensured that the muscle stretch speed was always reflected in the system faster than the force load.

As a solution to these newly discovered issues, a bio-inspired co-contraction mechanism was introduced into the system to regulate the post-reflex behavior of the Ia reflex.

### 3.4 Performance experiments of the complete double-reflex system

**FIGURE 9:**
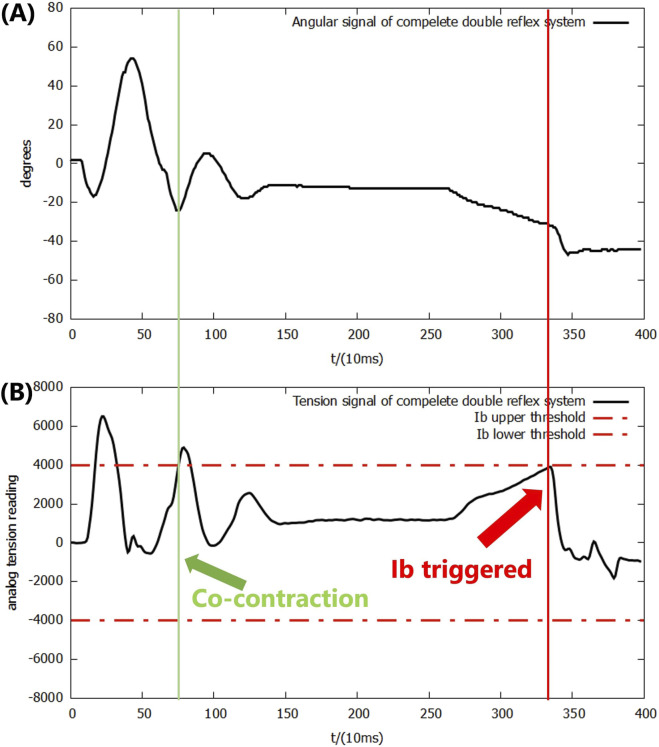
**(A)** Angular signal of the double-reflex system with mutex lock and co-contraction. **(B)** Analog tension signal of the double-reflex system with mutex lock and co-contraction.

According to Figure 9, the system successfully achieved the designated motor task after the application of the co-contraction mechanism into the double-reflex system. Both upward and downward overshoots were decreased by the co-contraction, along with the second falling impact. In addition, the Ib reflex was no longer mis-triggered by the Ia reflex feedback.

Note that co-contraction not only reduces the oscillation caused by the Ia reflex but its own mutex lock time period also slightly elongated the overall mutex lock time of co-contraction, providing a longer wait time for the Ia reflex to settle before starting to listen for a new reflex. Even though they have the same threshold signal and similar working mechanisms, co-contraction was not integrated with the Ia reflex. Such a design allows co-contraction to work independently when dealing with smaller impacts that will not trigger the Ia reflex, and it can be easily disabled in other motor tasks where precise control is not needed.

The outcomes also show that the lever of the system does not actually return to the ideal 0-degree position. This is caused by the elasticity of the materials in the McKibben PAM; the muscle was deformed by the gravitational force of the mass even when the pressure in both muscles was exactly 0.5.

The data from the trial were then analyzed through a side-by-side comparison with a PID-only control system and with the double-reflex system with only mutex locks (referred to as reflexes + mutex) in order to evaluate the quantitative performance improvement introduced by the co-contraction mechanism. The efficiency of the mechanism was evaluated in two separate phases of the experiment: co-contraction feedback only and Ia reflex feedback. Since co-contraction was not implemented in the earlier experiments, a simple PID-only system was used to evaluate its performance when triggered alone. Note that the Ib feedback period was not analyzed for two reasons: first, the Ib reflex from the reflexes + mutex system was mis-triggered immediately after the Ia reflex feedback, making it impossible to define a proper starting point for calculating the settling time because it overlapped with the Ia feedback period; second, the post-Ib reflex behavior is not regulated by the co-contraction mechanism under the current priority scheme and motor-task design; as described in Section 3.3, once triggered, the basket simply drops to the ground.

**FIGURE 10:**
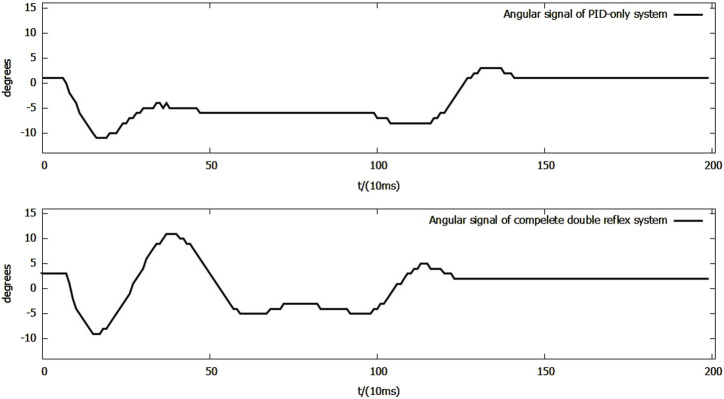
Side-by-side comparison of angular signals of the PID-only system and the complete double-reflex system in the co-contraction feedback-only scenario.

When co-contraction is triggered alone, as shown in Figure 10, the co-contraction mechanism behaves like a weaker version of the Ia reflex, demonstrating similar advantages and disadvantages. The settling time of the system was reduced by 180 ms, while the maximum downward overshoot decreased by 2°, and an upward overshoot with a maximum angle of 11° was produced by the feedback.

**FIGURE 11:**
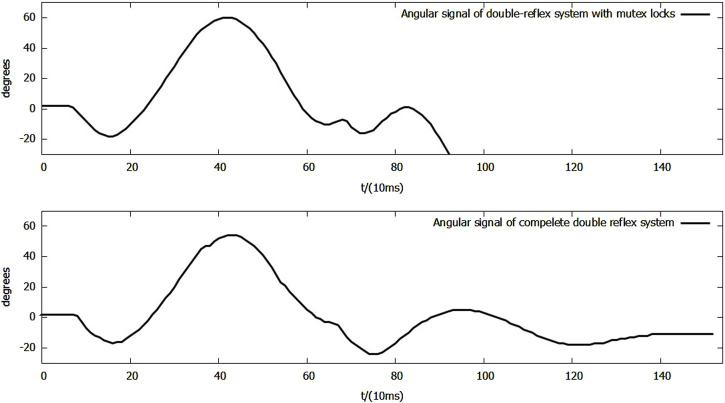
Side-by-side comparison of angular signals of reflexes + mutex system and complete double-reflex system during Ia feedback.

During the Ia reflex feedback period shown in Figure 11, the maximum downward overshoot decreased from −18° to −17°, while the maximum upward overshoot decreased from 60° to 54°, and the settling time of Ia feedback in the complete double-reflex system was 1,390 ms. The settling time of the double-reflex system with only mutex locks could not be calculated because the Ib reflex was mis-triggered as the lever fell.

The contraction range of the used McKibben PAMs was relatively narrow for all three feedbacks to function. The current co-contraction feedback only operates in its minimum capacity: properly separating the Ia reflex and the Ib reflex. With a wider contraction range, it is expected that the co-contraction mechanism can provide greater stability to the system than the current results.

## 4 Conclusions

In this study, a comprehensive exploration was conducted to realize the integration of Ia and Ib reflexes in a PAM-driven musculoskeletal system. The single-DOF musculoskeletal system presented by previous research was modified into a dual-reflex system capable of implementing both Ia and Ib reflexes simultaneously. After incorporating the necessary mechanisms to achieve multiple reflexes in a single trial, the initial “raw” integration of Ia and Ib reflexes was tested. The experimental results indicated that mis-triggering between the Ia and Ib reflexes was the main obstacle in achieving a stable dual-reflex system in a robotic context.

To address this issue, a mutex feedback lock was implemented to enhance system stability. Although this mechanism prevented mid-feedback mis-triggering, the post-reflex behavior of the Ia reflex still induced oscillations that could mis-trigger unwanted reflexes after the feedback period. As a solution, a co-contraction mechanism was introduced and tested. The experimental outcomes revealed that the system, equipped with all the introduced mechanisms, successfully achieved the desired dual-reflex functionality.

Nevertheless, the system remains insufficient as a foundation for a universal model of reflex control system design as it was tailored for a specific task. For example, post-Ib reflex behavior may also need regulation during certain reaching movements to avoid mis-triggering the Ia reflex when the Ib reflex does not drop obstacles directly to the ground. With a high-frequency, multi-threaded system and sensors offering higher sensitivity and precision, reflex feedback could be calculated in a more dynamic and precise manner rather than relying on the Max tracker, which would enhance the system’s performance.

Despite the challenges faced, the proposed dual-reflex control system provides valuable insights into the practical construction of reflex-controlled robots and suggests possible mechanisms that might exist in the human body.

## Data Availability

The original contributions presented in the study are included in the article/Supplementary Material; further inquiries can be directed to the corresponding author.
